# Dr. Sachs’s Death

**Published:** 1916-09

**Authors:** 


### Dr. Sachs's Death.

---

The untimely death of Dr. Theodore B. Sachs gives opportunity for some general remarks about the selfishness and jealousies of men who should be above such trivial things. Physicians, of all men, should be big brained and generous in their dealings with their fellow men, and especially with other physicians. It matters not how Sachs died, but it is known that in all his efforts for the amelioration of the suffering of those afflicted with tuberculosis, and in his labors to combat the disease, he received far more criticism than encouragement. He was derided, abused, misrepresented, and misunderstood by the very men who should have stood by and helped him. He may have been a man with some faults—he was called ambitious—but who has not grievous shortcomings? He may have sought to advance himself, but he used whatever self-advancement he obtained for the good of others. Certainly men are justified in seeking reputation and strength if those things are to be used by them in making the world a better place in which to live. Sachs gave all that he had, all that he made, that others might suffer less. He died poor in everything except big hearted sympathy with his fellow men.

Had he received the loyal support of his fellow physicians there is no estimating what he might have accomplished, for he did wonders when almost the entire profession was fighting him. He was opposed because of his great work, because he was in advance of those about him. Had he been satisfied to go along taking what little practice came to him, antagonizing the views of no one, and without any views of his own worth considering, he would have had an easier life, and might have died in obscure and useless wealth. He preferred to try with all his might to help others.

Chicago needed him, the world needed him, the medical profession needed him, and he might be living today to have given many more years of a useful life to the world and to the profession had only the helping hand and the encouraging word been extended him.

The inhumanity of man accounts for many wrongs; physicians are but men, but they are men of superior education, of higher ideals, of nobler motives than most men have; at least they should have these characteristics. Instead of attempting to destroy each other, they should work for the general good of the profession, and through the profession for the general good of the public. In serving others they serve themselves, in helping a fellow physician in his laudable efforts they are multiplying their own talents and doing the world a service of good deeds.

Sachs has passed to his reward, but there are other men who need encouragement just as much as did Sachs; there are others struggling just as hard in unselfish efforts. Why not give them the aid they deserve; and be quicker to do this than to impute bad motives and to criticise?

